# Association between high adolescent smartphone use and academic impairment, conflicts with family members or friends, and suicide attempts

**DOI:** 10.1371/journal.pone.0219831

**Published:** 2019-07-15

**Authors:** Min-Hyuk Kim, Seongho Min, Joung-Sook Ahn, Chisoo An, Jinhee Lee

**Affiliations:** 1 Department of Psychiatry, Yonsei University Wonju College of Medicine, Wonju, Korea; 2 Division of Child and Adolescent Psychiatry, Yonsei University Wonju College of Medicine, Wonju, Korea; Catholic University of Korea College of Medicine, REPUBLIC OF KOREA

## Abstract

This study aims to evaluate the association between smartphone use and suicide attempts, independent of possible confounders, including conflicts with family/friends and poor academic performance due to smartphone use. Data were obtained from the 2017 Korea Youth Risk Behavior Web-based Survey, a nationally representative survey of middle- and high-school students (N = 62,276). Time spent using a smartphone was divided into four categories: less than 1 h, 1–2 h, 3–4 h, and 5 h or more a day. The association of conflicts with family due to smartphone use, conflicts with friends due to smartphone use, and poor academic performance due to smartphone use with suicide attempts and time spent using a smartphone were analyzed using multiple and binary logistic regression analyses, respectively. The relationship between time spent on a smartphone and suicide attempts was analyzed using a multiple logistic regression analysis. All analyses were also stratified according to the main purpose of smartphone use (process purposes/social purposes). Conflicts with family/friends due to smartphone use was significantly associated with suicide attempts (P <0.001). The variables of conflicts with family, conflicts with friends and poor academic performance were also proportionally related to higher smartphone use (P <0.001). The use of a smartphone was significantly associated with suicide attempts in a multiple logistic regression analysis (adjusted odds ratio for smartphone use 5 h or more a day 2.16; 95% CI 2.07–2.26; P <0.001), and the association was more prominent with smartphone use for process purposes. Conflicts with family, conflicts with friends, poor academic performance, and suicide attempts were related to higher smartphone use in Korean adolescents. Time spent on a smartphone was positively related to suicide attempts, even after adjusting for conflicts with family members or friends and poor academic performance due to smartphone use.

## Introduction

The smartphone has become the most frequently used technology worldwide and is seen as a necessity in modern society [1]. Due to the multifunctionality of smartphones, they have become essential information and communication tools of daily life [2]. Smartphone ownership has been growing rapidly worldwide; for example, the rate in the US increased from 35% in 2011 to 64% in 2015 [3], and smartphone ownership among South Koreans in 2016 was 88%, which is the highest in the world [1]. Particularly among adolescents, who are in the forefront of new technology and media use [4], smartphones have become an important part of life. Some 97% of adolescents in Switzerland [5] and 84% of adolescents in Japan [6] have reported having their own smartphone.

Adolescents can be more vulnerable to the adverse effects of smartphone use because they are uncritically receptive and easily adapt to new technologies. In addition, they are sensitive to the influence of their environment and peers. A study by Jo et al. found that adolescents are vulnerable to smartphone addiction, which is similar to substance addiction and other types of behavioral addiction [7]. For example, 60% of UK adolescents have admitted that they are highly addicted to their smartphones [6], and according to a government survey in South Korea the smartphone addiction rate among adolescents was 18%, double the 9.1% addiction rate for adults [6,8]. In another study, the percentage of adolescents with addictive smartphone usage was 80%, whereas the rate was 58% among adults [9]. A prior study has indicated that smartphone usage time is closely related to smartphone addiction and provided better indicators for smartphone addiction than any others (i.e., use frequency, time until first smartphone use in the morning) [5]. Furthermore, some studies suggest that excessive smartphone use by adolescents, who are vulnerable to emotional changes and interpersonal issues, could be associated with various psychopathologies and behavioral problems [10–12].

Smartphones are useful for multiple purposes, including general productivity enhancement, information seeking, social interaction, diversion, relaxation, and entertainment [13]. Smartphones also provide enhanced educational productivity [14]. However, numerous studies have indicated that although smartphones can be useful, their overuse can result in a range of problems that are similar to internet overuse. Excessive smartphone use has been related to significant risks to well-being [15], and it also has adverse effects on physical [16,17] and mental health [11]. Excessive use of smartphones is also associated with decreased productivity and is negatively related to students' academic performance [18]. Specifically, excessive smartphone use has been associated with various psychopathologies and behavioral problems in adolescents. In a study on university students, the authors found that certain psychological characteristics, such as depression, anxiety, type A personality, high stress levels, and low mood might predispose students to excessive smartphone use [10]. The results of another study focusing on young adults suggested a positive relationship between excessive smartphone use and stress, a negative relationship between excessive smartphone use and academic performance, and a mediated negative relationship between excessive smartphone use and life satisfaction [18]. Despite the possible associations between excessive smartphone use and mental health outcomes, few studies have specifically examined its relationship to suicidal behaviors, one of the most important and critical factors in adolescent mental health.

In our study, we used a school-based, nationally representative data set of the Korean adolescent population to investigate the association between time spent on a smartphone and suicide. The running hypothesis of this study was that spending more time on a smartphone might increase suicide attempts. This study also assumed that conflicts with family, conflicts with friends, and poor academic performance were linked with both smartphone use and suicide because interpersonal issues and academic burden are among the most important of risk factors for adolescent suicide attempt [19–22].

## Method

### Study population and data collection

Data on the study population were obtained from the 13th Korea Youth Risk Behavior Web-based Survey (KYRBS), which was administered in 2017 by the Korean Ministry of Education, Science and Technology, the Ministry of Health and Welfare, and the Korea Centers for Disease Control and Prevention. The KYRBS is a nationally representative sample of Korean adolescents (aged 12–18 years) that originally included over 123 questions in 13 domains of health-risk behaviors [23]. To select a representative sample, a multi-stage clustered probability design based on the administrative district and school grade (middle and high school) was adopted. In the 13th KYRBS, a total 62,276 (95.8% response rate) students from 799 schools (99.8% response rate) responded to the survey [24]. Written informed consent was obtained from each participant prior to the survey. All the data used in this study were fully anonymized before access and were analyzed anonymously. This consent procedure was approved by the Institutional Review Board of the Korea Centers for Disease Control and Prevention (2014-06EXP-02-P-A).

### Measures

The exposure variable, *the number of hours spent using a smartphone*, was assessed by the question “On an average school day, how many hours do you use a smartphone?” Participants were divided into four groups according to the time spent in smartphone use: (1) less than 1 h; (2) 1–2 h; (3) 3–4 h; and (4) 5 h or more a day [5]. We also adopted the concepts proposed in previous studies to identify and categorize the main purpose of using a smartphone [25]. From the question, “In the last 30 days, please select only one service that you used mainly, when using your smartphone,” 13 answers were possible, and the answers were classified into two categories: (1) process purposes (e.g., studying, information retrieval, games, watching movies, reading comics and fiction, listening to music, using User-Created Contents (UCC) and videos, and online shopping) and (2) social purposes (e.g., messaging and chat, communities, e-mail, and social networks). From the question, “In the last 30 days, have you ever experience the severe conflicts with family due to your smartphone usage?” and “In the last 30 days, have you ever experience the severe conflicts with friends due to your smartphone usage?”, participants were asked whether they had experienced any conflicts with family and conflicts with friends due to smartphone use (Yes/No), which would suggest a tolerance that is one of the important factors of smartphone addiction [26]. They were also asked whether they had experienced poor academic performance due to smartphone use (Yes/No), by the question “In the last 30 days, were there any difficulties in your academic performance due to your smartphone usage?”, which would suggest a daily disturbance due to smartphone addiction [26].

The outcome variable, *suicide attempt*, was assessed by the question “During the past 12 months, did you ever attempt suicide?” Participants responded with one of the following: (1) No, I never attempted suicide; or (2) Yes, I have attempted suicide at least once.

For the control variables, the sociodemographic and general characteristics employed included age, sex, residential area, family economic status, hours of sleep, subjective stress, current alcohol consumption, cigarette smoking, and violence, all of which the literature has linked to suicidal behaviors. Respondents who lived in the country or in rural areas were categorized as “rural area”; those who lived in small- to middle-sized areas were categorized as “small city”; and those who lived in large cities were categorized as “large city”. Family economic status was assessed by the question, “What is your family's economic status?” The five possible response categories (very high; high; middle; low; and very low) were combined into three subgroups: high (very high or high); middle (middle); and low (very low or low) [27]. Sleep hours were divided into two categories: under 6 h; and 6 or more hours. We also evaluated the subjective stress level of the participants by asking “How much stress do you usually feel?” The five possible response categories were the same as the five noted above. Participants were also asked about their current alcohol consumption and cigarette smoking (Yes/No). To measure violence, we used the question: “Have you had any experience of receiving treatment at a hospital for violence (being physically assaulted, threatened, or bullied) from friends, superiors?” (Yes/No).

### Statistical analysis

The participants' characteristics according to each quartile of smartphone use were summarized using either a one-way analysis of variance for continuous variables or a chi-squared test for categorical variables with Bonferroni correction. We also performed a chi-squared test and a binary logistic regression analysis to investigate the proportion of responses to the main purpose of smartphone use for each quartile.

To analyze the associations between the adolescents' smartphone use and its adverse consequences, univariate logistic regression tests were performed using “smartphone use” as the principal predictor and each adverse consequence (conflicts with family/friends and poor academic performance due to smartphone use) as the main outcome variables.

Given that adolescent suicide attempts can be influenced by a number of intertwined emotional problems such as interpersonal problems and academic stress [28,29], we extended the previous analyses to take into account the effects of smartphone use on suicidal behavior. We first analyzed smartphone usage regarding their association with conflicts with family, conflicts with friends, and poor academic performance, all of which can influence attempts at suicide. We then analyzed the association between smartphone use and suicide attempts through multivariable logistic regression, adjusting for conflicts with family, conflicts with friends, and poor academic performance, along with other covariates.

Finally, the odds ratios (ORs) for suicide attempts in terms of conflicts with family, conflicts with friends, and poor academic performance due to smartphone use were calculated using multiple logistic regression analyses with complex sampling, adjusting for age, sex, residential area, family economic status, hours of sleep, subjective stress, current alcohol consumption, cigarette smoking, and violence. Considering the different association between psychosocial risk factors and mental health outcomes with respect to gender in previous study, the analysis were assessed after stratifying the subjects by gender [30,31].

All the ORs were also stratified by the main purpose of smartphone use. Two-tailed analyses were conducted, and P-values lower than 0.05 were considered significant. Adjusted ORs (AORs) and 95% confidence intervals (CIs) were calculated. All the statistical analyses were performed using SPSS software (version 23.0, IBM Corp., Armonk, NY, USA).

## Results

Table 1 shows the descriptive characteristics according to the quartiles of length of smartphone use. The participants used a smartphone for a mean 180.12 min per day. The results showed the proportion of girls, living in a large city, with middle-to-low family economic status, with less than 6 h of sleep, with high subjective stress levels, with current alcohol consumption, with current cigarette smoking, and having experienced violence, became significantly higher as the quartile of smartphone use time increased (all P < 0.001).

**Table 1:** General characteristics of participants according to time spent using a smartphone.

Characteristics		Smartphone use time					t/x or F	P-value
		Total sample	<1 h	1–2 h	3–4 h	≥5 h		
		N = 62,276 (%)	N = 18,926 (%)	N = 15,526 (%)	N = 16,233 (%)	N = 11,591 (%)		
Age							30.36	<0.001*
	Mean (SD)	15.00 (1.75)	14.98 (1.79)	14.91 (1.76)	15.03 (1.71)	15.10 (1.71)		
Sex							1494.22	<0.001†
	Boys	50.8	60.9	52.3	44.7	40.7		
	Girls	49.2	39.1	47.7	55.3	59.3		
Region							84.79	<0.001†
	Rural area	7.8	8.1	6.3	7.5	9.5		
	Small city	44.4	45.1	46.7	43.7	40.9		
	Large city	47.9	46.7	47.0	48.7	49.7		
Family economic status							840.94	<0.001†
	High	39.8	46.1	41.6	35.8	32.8		
	Middle	45.9	41.6	46.5	48.6	48.3		
	Low	14.3	12.2	11.9	15.6	18.9		
Sleep hours							47.44	<0.001†
	<6hrs	32.6	31.5	31.5	33.3	35.1		
	≥6hrs	67.4	68.6	68.5	66.7	64.9		
Subjective stress level							509.25	<0.001†
	High	37.3	34.4	34.3	38.8	44.1		
	Middle	42.2	41.8	44.1	42.9	39.4		
	Low	20.5	23.8	21.7	18.3	16.5		
Current alcohol consumption							1834.47	<0.001†
	No	76.2	79.4	77.4	73.9	72.6		
	Yes	23.8	20.6	22.6	26.1	27.4		
Current smoking							1174.79	<0.001†
	No	93.0	94.2	93.8	92.4	90.6		
	Yes	7.0	5.8	6.2	7.6	9.4		
Experience of violence							16.30	<0.001†
	No	75.7	76.6	75.2	76.0	74.7		
	Yes	24.3	23.4	24.8	24.0	25.3		

* One-way ANOVA test analysis, significance at P < 0.005.

† Chi-squared test with Bonferroni correction, significance at P < 0.005.

Table 2 shows the smartphone usage rates according to purposes of smartphone. The time spent on social purposes was greater than that spent on process purposes. In particular, the girls were more likely to use the smartphones for social purposes at all usage levels, whereas the boys used the smartphones more for social than process purposes only when their usage was 5 or more hours a day. The ORs for using the smartphone for social purposes were 1.73 (1.71–1.75) and 2.77 (2.74–2.81) in boys and girls, respectively. Because the purpose for using the smartphone was related to the time spent on the device, we performed a sub-analysis including the purpose.

**Table 2:** Main purpose of smartphone use according to spent time using a smartphone.

Main purpose	Smartphone use time					t/x or F	P-value
	Total sample	< 1 h	1–2 h	3–4 h	≥5 h		
	N = 62,276 (%)	N = 18,926 (%)	N = 15,526 (%)	N = 16,233 (%)	N = 11,591 (%)		
Total sample						1222.71	<0.001*
Process purposes	40.5	53.2	49.2	36.9	31.4		
Social purposes	59.5	46.6	57.1	63.1	68.6		
OR for social purposes (95% CI)		Ref	1.51 (1.50–1.52)	1.94 (1.93–1.95)	2.46 (2.44–2.48)		<0.001†
Boys						226.50	<0.001*
Process purposes	53.2	60.3	54.1	50.3	46.8		
Social purposes	46.8	39.7	45.9	49.7	53.2		
OR for social purposes (95% CI)		Ref	1.31 (1.30–1.32)	1.51 (1.49–1.52)	1.73 (1.71–1.75)		<0.001†
Girls						643.15	<0.001*
Process purposes	28.4	42.8	30.7	26.0	20.7		
Social purposes	71.6	57.2	69.3	74.0	79.3		
OR for social purposes (95% CI)		Ref	1.63 (1.61–1.64)	2.09 (2.07–2.81)	2.77 (2.74–2.81)		<0.001†

* Chi-squared test with Bonferroni correction, significance at P < 0.005.

† Univariable logistic regression analysis for smartphone use for social purposes.

Adverse consequences of smartphone use, including conflicts with family, conflicts with friends, and poor academic performance, showed significant associations with the amount of spent time on the smartphone in the logistic regression analyses (Table 3). In both boys and girls, the associations were more prominent in smartphone use for process purposes.

**Table 3:** Univariable logistic regression analysis of conflicts with family, conflicts with friends, and poor academic performance due to smartphone use, according to time spent using smartphone.

		Total sample			Boys			Girls		
		OR	95% CI	P-value	OR	95% CI	P-value	OR	95% CI	P-value
Conflicts with family due to smartphone use										
Total				<0.001*			<0.001*			<0.001*
	<1 h	1			1			1		
	1–2 h	1.57	1.49–1.65		1.53	1.43–1.63		1.59	1.48–1.72	
	3–4 h	1.77	1.69–1.86		1.65	1.54–1.76		1.86	1.73–2.00	
	≥5 h	1.76	1.67–1.85		1.51	1.41–1.63		1.92	1.78–2.07	
Process purposes				<0.001*			<0.001*			<0.001*
	<1 h	1			1			1		
	1–2 h	1.59	1.49–1.71		1.59	1.46–1.73		1.62	1.43–1.82	
	3–4 h	1.88	1.76–2.02		1.79	1.64–1.96		2.06	1.83–2.33	
	≥5 h	1.76	1.62–1.91		1.60	1.44–1.77		2.08	1.82–2.39	
Social purposes				<0.001*			<0.001*			<0.001*
	<1 h	1			1			1		
	1–2 h	1.53	1.43–1.63		1.50	1.36–1.65		1.51	1.38–1.66	
	3–4 h	1.66	1.56–1.77		1.52	1.38–1.68		1.68	1.54–1.84	
	≥5 h	1.68	1.57–1.80		1.45	1.30–1.61		1.74	1.58–1.91	
Conflicts with friends due to smartphone use										
Total				<0.001*			<0.001*			<0.001*
	<1 h	1			1			1		
	1–2 h	1.36	1.28–1.45		1.37	1.27–1.48		1.43	1.29–1.58	
	3–4 h	1.63	1.53–1.73		1.58	1.46–1.71		1.83	1.66–2.01	
	≥5h	2.09	1.97–2.22		1.85	1.70–2.01		2.54	2.31–2.80	
Process purposes				<0.001*			<0.001*			<0.001*
	<1h	1			1			1		
	1–2 h	1.47	1.35–1.61		1.47	1.33–1.63		1.52	1.29–1.79	
	3–4 h	1.70	1.56–1.85		1.68	1.51–1.86		1.89	1.61–2.21	
	≥5 h	2.11	1.92–2.33		1.92	1.71–2.16		2.71	2.29–3.22	
Social purposes				<0.001*			<0.001*			<0.001*
	<1 h	1			1			1		
	1–2 h	1.25	1.15–1.35		1.26	1.13–1.41		1.30	1.15–1.46	
	3–4 h	1.52	1.40–1.64		1.47	1.31–1.64		1.66	1.48–1.85	
	≥5 h	1.96	1.81–2.13		1.74	1.54–1.96		2.25	2.01–2.52	
Poor academic performance due to smartphone use										
Total				<0.001*			<0.001*			<0.001*
	<1 h	1			1			1		
	1–2 h	1.70	1.62–1.78		1.62	1.51–1.72		1.70	1.58–1.84	
	3–4 h	1.98	1.88–2.08		1.78	1.67–1.91		1.97	1.83–2.12	
	≥5 h	1.92	1.82–2.02		1.71	1.58–1.84		1.88	1.74–2.03	
Process purposes				<0.001*			<0.001*			<0.001*
	<1 h	1			1			1		
	1–2 h	1.7	1.62–1.86		1.73	1.59–1.88		1.74	1.54–1.96	
	3–4 h	2.03	1.89–2.18		1.93	1.76–2.11		2.12	1.89–2.39	
	≥5 h	1.89	1.74–2.05		1.82	1.64–2.02		1.92	1.67–2.20	
Social purposes				<0.001*			<0.001*			<0.001*
	<1 h	1			1			1		
	1–2 h	1.64	1.54–1.75		1.52	1.38–1.68		1.64	1.49–1.80	
	3–4 h	1.86	1.75–1.99		1.65	1.50–1.82		1.83	1.67–2.01	
	≥5 h	1.81	1.69–1.94		1.59	1.43–1.77		1.75	1.59–1.92	

* OR: odds ratio; significance at P < 0.005.

Independent variables, such as conflicts with family due to smartphone use (AOR 1.41; 95% CI 1.24–1.61; P <0.001) and conflicts with friends due to smartphone use (AOR 1.50; 95% CI 1.32–1.70; P <0.001) were positively associated with suicide attempts; however, poor academic performance due to smartphone use was not (Table 4).

**Table 4:** Multivariable logistic regression analyses of conflicts with family, conflicts with friends, and poor academic performance due to smartphone use (independent variables) for suicide attempt (dependent variable).

		Total		Boys		Girls	
		AOR^a^	95% CI	AOR^a^	95% CI	AOR^a^	95% CI
Conflicts with family members due to smartphone use							
Total	No	1		1		1	
	Yes	1.41	1.24–1.61	1.27	1.02–1.58	1.49	1.26–1.76
Process purposes	No	1		1		1	
	Yes	1.17	0.95–1.44	1.18	0.89–1.57	1.14	0.85–1.53
Social purposes	No	1		1		1	
	Yes	1.46	1.34–1.86	1.43	1.03–2.00	1.65	1.35–2.02
Conflicts with friends due to smartphone use							
Total	No	1		1		1	
	Yes	1.50	1.32–1.70	1.94	1.47–2.56	1.39	1.16–1.66
Process purposes	No	1		1		1	
	Yes	1.56	1.26–1.92	1.92	1.45–2.55	1.20	0.87–1.66
Social purposes	No	1		1		1	
	Yes	1.49	1.27–1.74	1.50	1.08–2.07	1.47	1.23–1.77
Poor academic performance due to smartphone use							
Total	No	1		1		1	
	Yes	1.00	0.88–1.13	1.10	0.89–1.36	0.95	0.81–1.11
Process purposes	No	1		1		1	
	Yes	1.00	0.82–1.23	1.14	0.86–1.52	0.88	0.66–1.18
Social purposes	No	1		1		1	
	Yes	0.99	0.84–1.16	1.02	0.74–1.40	0.98	0.82–1.18

AOR: adjusted odds ratio.

^a^ Adjustment for age, sex, region of residence, family economic status, subjective stress, sleep hours, current alcohol consumption, current smoking, experience of violence, and time using a smartphone.

Therefore, the conflicts with family and conflicts with friends due to smartphone use were adjusted to analyze the association between time spent on a smartphone and suicide attempts. Longer time on a smartphone was significantly associated with suicide attempts in the multivariable logistic regression analyses (Table 5). Adolescents reporting 3–4 h (AOR 1.09; 95% CI 1.06–1.12) and 5 h or more (AOR 1.79; 95% CI 1.74–1.84) of daily smartphone use were significantly more likely to report a suicide attempt over the previous year compared with adolescents reporting less than 1 h of daily use. In adolescents who used a smartphone for process purposes, all levels from 1–2 to 5 h or more of smartphone use were related to suicide attempts (1–2 h, AOR 1.31, 95% CI 1.25–1.32; 3–4 h, AOR 1.32, 95% CI 1.26–1.38; 5 h or more, AOR 2.16, 95% CI 2.07–2.26) compared with less than 1 h. However, in adolescents who used a smartphone for social purposes, 1–2 h of smartphone use was shown to be protective against suicide attempts, with an AOR of 0.83 (95% CI 0.79–0.86). The subgroup analyses by sex also showed comparable results in each logistic analysis.

**Table 5:** Subgroup analysis of adjusted odd ratios for time spent using a smartphone and suicide attempt.

		Total			Boys			Girls		
		AOR^a^	95% CI	P	AOR^b^	95% CI	P	AOR^b^	95% CI	P
Total				<0.001*			<0.001*			<0.001*
	<1 h	1			1			1		
	1–2 h	0.98	0.95–1.01		0.97	0.93–1.01		0.99	0.95–1.03	
	3–4 h	1.09	1.06–1.12		1.22	1.17–1.28		1.02	0.98–1.06	
	≥5 h	1.79	1.74–1.84		1.62	1.54–1.69		1.81	1.74–1.87	
Process purposes				<0.001*			<0.001*			<0.001*
	<1 h	1			1			1		
	1–2 h	1.31	1.25–1.36		1.22	1.16–1.30		1.39	1.31–1.48	
	3–4 h	1.32	1.26–1.38		1.32	1.25–1.40		1.32	1.24–1.41	
	≥5 h	2.16	2.07–2.26		2.02	1.90–2.14		2.33	2.18–2.48	
Social purposes				<0.001*			<0.001*			<0.001*
	<1 h	1			1			1		
	1–2 h	0.83	0.79–0.86		0.75	0.70–0.81		0.87	0.82–0.91	
	3–4 h	1.00	0.97–1.04		1.18	1.11–1.26		0.95	0.91–1.00	
	≥5 h	1.65	1.59–1.71		1.38	1.29–1.47		1.65	1.61–1.76	

* AOR: adjusted odds ratio; significance at P < 0.005.

^a^ Adjustment for age, sex, region of residence, family economic status, subjective stress, sleep hours, current alcohol consumption, current smoking, experience of violence, conflicts with family, conflicts with friends, and poor academic performance.

^b^ Adjustment for age, region of residence, family economic status, subjective stress, sleep hours, current alcohol, current smoking, experience of violence, conflicts with family, conflicts with friends, and poor academic performance.

## Discussion

This study examined the association of smartphone use and suicide attempts within a relatively large convenience sample of adolescents in Korea. Four key factors associated with smartphone use were assessed. First, the main purposes of smartphone use are as follows: process purposes or social purposes determined the adolescents' levels of use. Among both boys and girls, high smartphone use was associated with more time using the smartphone for social purposes. Second, conflicts with family, conflicts with friends and poor academic performance were significantly related to greater use of a smartphone. Furthermore, both boys and girls were more likely to show adverse effects from using smartphones for process purposes, and girls were found to be more prone to adverse consequences from smartphone overuse compared with boys, regardless of the main purpose for use. Third, smartphone overuse and conflicts with family and conflicts with friends related to smartphone use predicted suicidal behaviors, however, poor academic performance due to smartphone use did not. In particular, both girls having family conflicts due to using a smartphone for social purposes and boys conflicting with friends due to using a smartphone for process purposes showed a higher correlation with suicide attempts. Finally, after adjusting for all the factors listed above, there was a significant association between time spent on a smartphone and suicide attempts among both boys and girls. The relevance was greater when adolescents used smartphones for process purposes. We also found a potential protective effect from moderate use (1–2 h) of a smartphone for social purposes with regard to suicide attempts.

The results of this study are largely consistent with the findings of previous studies. In terms of the potential problematic role of smartphone use in promoting adolescents' negative affect and psychopathologies, a previous systematic review by Elhai et al. reported on associations between excessive smartphone use and both depression and anxiety severity, and they also found some support for increased stress and reduced self-esteem [11]. A prior longitudinal study has suggested that the rapid adoption of smartphone technology can have a markedly negative impact on adolescents' psychological well-being due to their spending more time on electronic communication and less time on nonscreen activities, such as in-person social interaction [32]. Our study indicated that the major problems of suicidal behavior as well as conflicts with family, conflicts with friends, and poor academic performance should be addressed in adolescents with high smartphone use.

In addressing the relationship between smartphone use and suicide attempts, the results of this study should be considered under the prevalent understanding of media screen use, including use of the internet. Most research focusing on similar topics has addressed the associations between internet overuse and psychological distress and suicidal behaviors. A nationally representative study of adolescents in the US found an association between excessive internet use and risks for adolescent suicide [33]. Another study that had been implemented within the framework of the European Union project, with a sample of 11,356 school-based adolescents, indicated a correlation between problematic internet use and suicidal behaviors [34]. Most smartphone use is internet based, and addictive use can show many similarities to internet addiction [35], however, there are also some unique characteristics of smartphone such as ubiquity and immediacy. Due to the nature of the smartphone's easy and direct communication [26], it has been reported that smartphone addiction has a greater association with a desire for interpersonal relationships and is less related to features of social introversion than internet addiction [36]. Indeed, in our study, adolescents had more adverse consequences from the use of smartphones for process purposes compared with social purposes, and the conflicts with family and conflicts with friends related to smartphone use predicted suicidal behavior.

The association between the time spent on a smartphone and suicide attempts might be partially influenced by conflicts with family and conflicts with friends due to smartphone use. The effects of high smartphone use on interpersonal relationships could contribute to poor mental health in adolescents. Social deficits and withdrawal are known to be associated with depression, and depressed individuals tend to become preoccupied with the nonsocial aspects of technology use [37]. A prior study on Korean adolescents had indicated a significant association between smartphone addiction and family dysfunction or poor family relationships [12], and a recent study has indicated that adolescent excessive smartphone use was negatively associated with friendship satisfaction [38]. Smartphones can reduce the quality of interpersonal relationships in the real world because it omits many social cues, such as “nonverbal cues,” and involves less control of disclosure [39]. There have also been reports of a significant association between smartphone use and poor social skills [40], which leads to poor mental health in adolescents. Consequently, smartphone use was proportionally related to social distress, and its association with suicide attempts might be partially influenced by stress caused by conflicts with others, as demonstrated in the present study.

Our results from the stratification analysis of the main purposes for using a smartphone confirmed that there is a greater association between suicide attempts and using a smartphone for process purposes compared with using it for social purposes. These findings are similar to a recent smartphone study that found a closer relationship between smartphones used for process purposes and anxiety severity compared with those used for social purposes [25]. Some researchers have also suggested that pleasurable experiences that potentially result from process purposes-oriented smartphone use might result in losing behavioral control [13]. The results indicated that further research is needed to assess the different aspects on the purpose of smartphone usage and to expand them as a preventive or a treatment tool for adolescent suicidal behavior.

A number of studies have suggested that behavioral addictions, such as gambling, internet usage, and gaming, might share neurobiological mechanisms with substance addiction and that they have similarities including urges and cravings [41,42]. Moreover, studies have shown that addictive smartphone use including excessive usage time, is related to impulsivity, and high impulsivity is a psychological risk factor for developing addiction to social networking sites among smartphone users [7,43–45]. Adolescence is an important period for brain development, and overuse of a smartphone during adolescence has been shown to decrease functional connectivity in the anterior insula and the primary motor cortex [46], altering higher executive functions that promote continued risk for developing substance and behavioral addiction, mood dysregulations, and impulsive behavior [47]. These negative neurological and psychological traits might contribute to aggravating risk behaviors in adolescents with high smartphone use, including suicide attempts. Future studies on excessive use of smartphone, psychopathologies, and addictive personality characteristics might provide important insights into addiction-related risk behaviors, including suicide attempts. Considering the increasing rate of smartphone use among adolescents in recent years, effective strategies are needed to prevent high smartphone use-related adverse mental health outcomes.

### Limitations and strengths

The present study has several limitations. First, due to the cross-sectional nature of national surveys, causal inferences based on the findings from this present study might not be valid. Longitudinal studies are necessary to further investigate the risk and protective factors of smartphone use for suicidal behavior. Second, the level of smartphone use was calculated by self-reported answers, and participants were grouped into quartiles by time on a smartphone rather than smartphone addiction scales. However, excessive smartphone use was validated as the most powerful independent predictor of smartphone addiction in the previous study [10]. In addition, longer smartphone use was found to be a significant predictor of smartphone addiction, which was diagnosed by the validated smartphone addiction scale [5]. Third and last, most variables in the study, including conflicts with family/friends and poor academic performance due to smartphone use, and suicide attempts, were surveyed on the basis of a self-reported questionnaire, which has inherent limitations regarding the validity of the data and the recall bias. Therefore, further studies not only with self-reported psychiatric pathologies but also with more precise evaluation and diagnosis are needed to provide stronger evidence of an association between smartphone use and suicidal behavior in adolescents. However, the strength of the present study included the use of a multilevel multinomial logistic modeling approach based on a nationally representative sample of Korean adolescents, who have the highest smartphone ownership rate in the world [1]. Considering the possibility of increased use of smartphones worldwide in the future, the results of the present study could be a cornerstone for future research on issues related to their use among adolescents. Additionally, to our knowledge, this is the first study to investigate the association between adverse consequences of smartphone overuse and suicide attempts.

## Conclusion

It is noteworthy that we confirmed a significant and specific relationship between time spent on a smartphone and the prevalence of attempted suicide, independent of other problems associated with smartphone use, in a nationally representative sample of adolescents in Korea, even when controlling for conflicts with family, conflicts with friends, and poor academic performance. In addition, our findings indicate that high smartphone use for process purposes ultimately turned out to have stronger associations with negative outcomes, whereas moderate smartphone use for social purposes showed some protection against attempted suicide. The results of this study suggest that consideration should be given to improving screening and prevention for mental health problems in adolescents with excessive use of smartphones. Further research is needed to understand the impact of specific length of time using a smartphone on the mental health of adolescents to develop preventive approaches and to help strengthen current smartphone use guidelines.
